# Danshen Decoction in the Treatment of Hyperlipidemia: A Systematic Review and Meta-Analysis Protocol of Randomized Controlled Trials

**DOI:** 10.1155/2022/2392652

**Published:** 2022-11-08

**Authors:** Mengnan Liu, Xu Yanneng, Gang Yang, Ziyi Li, Gang Luo, Sijin Yang

**Affiliations:** ^1^Faculty of Chinese Medicine and State Key Laboratory of Quality Research in Chinese Medicine, Macau University of Science and Technology, Macau SAR 999078, China; ^2^National Traditional Chinese Medicine Clinical Research Base and Department of Cardiovascular Medicine, The Affiliated Traditional Chinese Medicine Hospital of Southwest Medical University, Luzhou 646000, China; ^3^Institute of Integrated Chinese and Western Medicine, Southwest Medical University, Luzhou 646000, China; ^4^Department of Interventional Radiology, The Affiliated Traditional Chinese Medicine Hospital of Southwest Medical University, Luzhou 646000, China; ^5^School of Clinical Medicine, Southwest Medical University, Luzhou 646000, China

## Abstract

*Background*. Hyperlipidemia is a common clinical chronic disease that increases the incidence of cardiovascular disease. However, although oral drug therapy can reduce blood lipids, long-term drug treatment may cause various side effects. Therefore, it is important to find suitable alternatives for the treatment of hyperlipidemia. The classic traditional Chinese medicine (TCM) prescription Danshen decoction (DSD) has been found effective for the treatment of hyperlipidemia. This protocol aims to evaluate the efficacy and safety of DSD in the treatment of hyperlipidemia. *Methods and Analysis*. We will screen all the randomized controlled trials (RCTs) which research DSD in the treatment of hyperlipidemia from 7 databases from their inception to July 2022; three investigators will independently screen and select RCTs and extract data and assess the risk of bias. The Cochrane scale, Jadad scale, and GRADE scale will be used to assess the risk of bias, literature quality, and outcome quality, respectively. Review Manager V.5.4 will be used for the meta-analysis, and the results will be presented as the risk ratio (RR) for the binary data and the mean difference (MD) or standardized mean difference (SMD) for the continuous data. *Ethical approval and Dissemination.* This protocol for a systematic review will be submitted to a peer-reviewed journal for publication and ethical approval is not applicable. *PROSPERO registration number.*CRD42022352467.

## 1. Introduction

Hyperlipidemia refers to high blood lipid levels, which can directly cause some diseases that seriously endanger human health, such as atherosclerosis, coronary heart disease, and pancreatitis [1]. Hyperlipidemia has been shown to be a major risk factor for the development of cardiovascular diseases (CVDs) [2]. In China, at least 41.9% of adults suffer from hyperlipidemia; a study indicates that by 2030 [3], there will be 9.2 million patients with CVDs caused by hyperlipidemia in China [4]. Early detection and treatment are essential to reduce cardiovascular events and premature death. Statins are the main treatment for hyperlipidemia [5]. However, long-term use of statins for lipid-lowering therapy will inevitably produce adverse effects on endocrine system and may lead to further increases in drug doses as drug resistance develops [6]. Therefore, a safe and effective alternative therapy or adjuvant therapy urgently needs to be proposed and applied.

Traditional Chinese medicine (TCM) has accumulated a lot of clinical experience in the treatment of hyperlipidemia, whether it is acupuncture, cupping, massage, moxibustion, or herbal medicine, and it is widely used in the treatment of obesity and hyperlipidemia [7]. At present, an increasing number of clinical studies have demonstrated the safety and efficacy of TCM in the treatment of hyperlipidemia [8–10], and these treatments can effectively downregulate blood lipid levels in patients, reduce body weight in obese patients, and improve clinical symptoms such as fatigue and drowsiness [11]. Danshen decoction (DSD) is a commonly used medicine for the treatment of CVDs, and it is composed of *Salvia miltiorrhiza* Bge., *Santalum album* L., and *Amomum villosum* Lour. Clinical studies have found that DSD is beneficial to the recovery of patients with hypertension, hyperlipidemia, heart failure, and coronary heart disease. More importantly, DSD as an adjuvant therapy for cardiovascular disease can better improve the clinical effective rate (CER) and reduce the occurrence of adverse reactions [12, 13]. Although studies have shown that DSD has application value in the treatment of cardiovascular diseases, there is still no systematic review to further verify the efficacy of DSD in the treatment of hyperlipidemia [14].

Overall, this meta-analysis protocol will address the lack of a systematic analysis of the efficacy and safety of DSD in the treatment of hyperlipidemia. Besides, we will categorize all randomized controlled trials (RCTs) of DSD in the treatment of hyperlipidemia, and electronic and manual searches will be used to perform a systematic review and meta-analysis [15–19]. Finally, we will discuss the efficacy and safety of DSD in the treatment of hyperlipidemia in order to provide evidence-based medicine for DSD in the treatment of hyperlipidemia in clinical decision-making.

## 2. Methods and Analysis

### 2.1. Protocol and Registration

This protocol has been registered on the PROSPERO platform (https://www.crd.york.ac.uk/PROSPERO/) with the registration number: CRD42022352467 [20], and this systematic review protocol complies with the Guidelines for the Preferred Reporting Items for Systematic Reviews and Meta-Analysis Protocol (PRISMA-P) statement. PRISMA-P checklist has been presented in the supplementary material (available here).

### 2.2. Eligibility Criteria

#### 2.2.1. Criteria for Inclusion and Exclusion

This systematic review and meta-analysis will include all RCTs of DSD alone or DSD combined with conventional treatment in the treatment of hyperlipidemia published in any language as of July 2022. These RCTs must include but are not limited to CER and various serological lipid indexes, including but not limited to total triglyceride (TG), total cholesterol (TC), low-density lipoprotein cholesterol (LDL-C), and high-density lipoprotein cholesterol (HDL-C), while observational studies, single case reports, literature reviews, etc., will not be included in the study.

Inclusion criteria are as follows:

- RCTs must be related to DSD.
- RCTs must be associated with hyperlipidemia.
- Patients included in the RCTs were diagnosed with hyperlipidemia, or met the diagnostic criteria for hyperlipidemia.
- Must include but not limited to CER and any one of the following indicators: TG, TC, LDL-C, and HDL-C.
- Quantitative indicators of hyperlipidemia must be included.

Exclusion criteria are as follows:

- The data in RCTs do not support a diagnosis of hyperlipidemia.
- The control group for the experimental group is unclear.
- The experimental data description is unclear, or the results are wrong.

#### 2.2.2. Types of Participants

This meta-analysis will include all patients who meet the diagnostic criteria for dyslipidemia in the Chinese Guidelines for the Prevention and Treatment of Dyslipidemia in Adults 2020 (TG ≥ 1.70 mg/L, TC ≥ 5.20 mmol/L, LDL-C ≥ 3.12 mmol/L, HDL-C ≤ 0.91 mmol/L, lipoprotein(a) (LP(a)) ≥ 300 mmol/L, apolipoprotein A1 (apoA1) ≥ 1.6 g/L, and apolipoprotein B (apoB) ≥ 1.6 g/L) [21–24]. In addition, RCTs need to use CER and any one of the serological lipid indicators as outcome indicators. There are no restrictions on age, gender, ethnicity, disease course, and disease severity [25].

#### 2.2.3. Types of Experimental Groups

In RCTs, the intervention of the experimental group refers to the use of DSD as an adjuvant or supplementary treatment on the basis of conventional treatment. The route of administration of DSD includes but is not limited to decoction, pills and powders, etc. The number of daily doses and doses are determined by the doctor according to the severity of the patient's condition and the drug metabolism function of the patient's gastrointestinal tract. In addition, it is not excluded that patients receive other nondrug TCM adjuvant treatments, including *Qigong*, *TaiChi*, acupuncture, moxibustion, and massage.

#### 2.2.4. Types of Control Groups

The intervention in the control group was defined as conventional treatment, mainly with hypolipidemic drugs, and other drugs were not excluded for the treatment of other underlying diseases and complications of patients.

### 2.3. Outcome Measures

#### 2.3.1. Primary Outcome

CER will be identified as the primary indicator for this meta-analysis.

#### 2.3.2. Secondary Outcome

Various serum lipid indexes will be identified as secondary indexes in this meta-analysis, including but not limited to TG, TC, LDL-C, and HDL-C.

### 2.4. Search Strategies for Data Sources

#### 2.4.1. Data Sources

Seven databases will be used for literature retrieval (PubMed (https://pubmed.ncbi.nlm.nih.gov), Web of Science (https://www.webofscience.com), Cochrane Library (https://www.cochranelibrary.com), China National Knowledge Infrastructure (CNKI) (https://www.cnki.net), Wanfang Database (https://www.wanfangdata.com.cn/), Chinese Science Journal Database (VIP Database) (http://lib.cqvip.com/), and China Biomedical Literature Database (CBM) (http://www.sinomed.ac.cn). The time for searching literature is from the establishment of the database to July 2022. The following keywords will be used for individual or combined searches: “Clinical Controlled Experiments,” “Clinical Observations,” “Danshen Decoction,” “Danshen Yin,” “Hypercholesterolemia,” “Hypertriglyceridemia,” “Blood Lipids,” “Triglycerides,” “Cholesterol,” “Hyperlipidemia,” “high-density lipoprotein cholesterol,” and “HDL-C,” “low-density lipoprotein cholesterol,” and all RCTs published in any language could be included. The search strategy of PubMed is shown in Table 1.

#### 2.4.2. Other Search Resources

Clinical trial databases such as China Clinical Trials Registry (ChiCTR) (https://ClinicalTrials.gov) will be searched for more data.

### 2.5. Data Collection and Screening

#### 2.5.1. Study Selection

**Figure 1:**
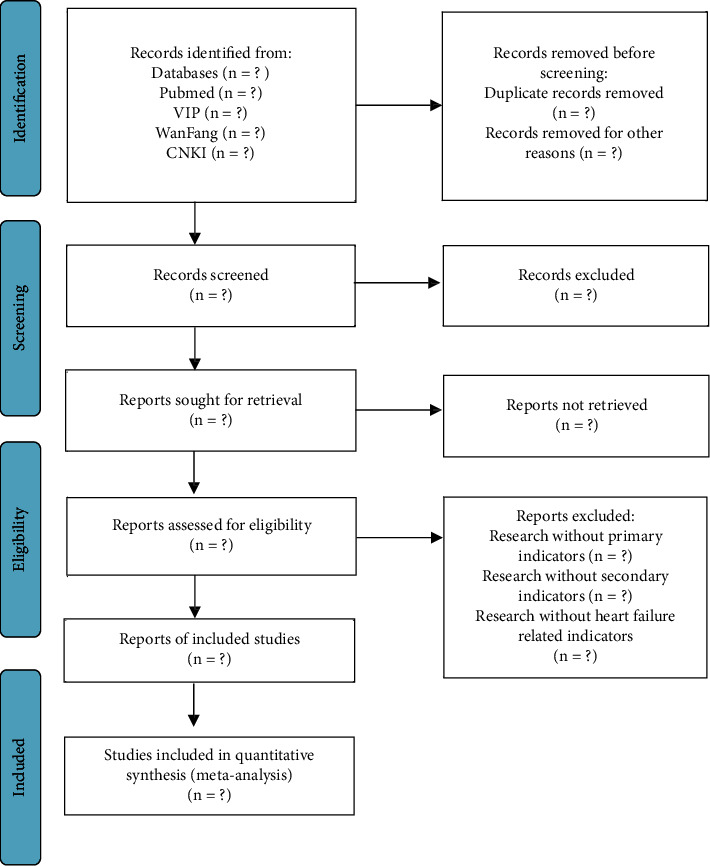
PRISMA flow diagram of the study selection process.

Two investigators (Mengnan Liu and Ziyi Li) will independently search the database and evaluate and screen RCTs for inclusion in the meta-analysis according to the inclusion-exclusion criteria, and any disagreements and discrepancies will be discussed with the third investigator (Yanneng Xu) to reach a consensus. The qualified RCTs finally screened will be imported into Zotero V.6.0.10 for sorting statistics. The flowchart of the study selection process is outlined in Figure 1.

#### 2.5.2. Data Extraction

Two investigators (Mengnan Liu and Ziyi Li) will independently extract data from eligible RCTs. For each RCT, the following information will be extracted: authors, year of publication, study design, treatment regimen, control intervention, sample size, characteristics of participants (age and sex, etc.), and primary and secondary outcome measures. If data are missing, where feasible, the corresponding authors of the study will be contacted for missing or incomplete data. Any disagreements and discrepancies will be discussed with the third investigator (Yanneng Xu) and the consensus will be reached.

### 2.6. Statistical Analysis

Review Manager V.5.4 software will be used for data statistics and analysis. Two investigators (Mengnan Liu and Ziyi Li) will independently organize and analyze the outcome indicators of RCTs, and multiple RCTs with the same outcome indicators will be jointly analyzed to consider the efficacy of DSD alone or DSD combined with conventional treatment on the outcome indicators. We will choose random- or fixed-effects models based on heterogeneity analysis.

### 2.7. Measures of Treatment Effect

Review Manager V.5.4 software will be used for data analysis, for outcomes we will choose relative risk (RR) to assess dichotomous outcomes, while for continuous outcomes we will use mean difference (MD) or standardized mean difference (SMD) to assess, and each outcome value will be presented with a 95% confidence interval (95% CI).

### 2.8. Risk of Bias

#### 2.8.1. Assessment of Risk of Bias

The Jadad scale will be used for the assessment of risk of bias, two investigators (Mengnan Liu and Ziyi Li) will independently use the Jadad scale to assess the bias score according to the actual situation of the RCTs [26], and articles will be classified as low, medium, or high risk of bias. Any disagreements and discrepancies will be discussed with the third investigator (Gang Yuan) and the consensus will be reached [27].

#### 2.8.2. Methodological Quality Assessment

The methodological quality of each included trial will be scored by two investigators (Mengnan Liu and Ziyi Li) according to the Cochrane collaboration tool [28]. It consists of seven domains: random sequence generation, allocation concealment, blinding of participants and personnel, blinding of outcome assessment, incomplete outcome data, selective reporting, and other biases. Three levels were used to assess the quality of the method: “low risk of bias” (+), “high risk of bias” (−), and “uncertain risk of bias” (?). If necessary, differences will be discussed with a third investigator (Gang Yuan) to reach an agreed conclusion.

### 2.9. Assessment of Publication Bias

If ten or more articles were to be included in this meta-analysis, funnel plots will be used to examine potential publication bias arising from an increase in the number of RCTs.

### 2.10. Dealing with Missing Data

If data are missing, where feasible, the corresponding authors of the study will be contacted in order to get the missing or incomplete data.

### 2.11. Heterogeneity Analysis and Subgroup Analysis

Chi-square tests and *I*^2^ tests will be used for heterogeneity analysis between RCTs, and if *I*^2^ > 50%, substantial heterogeneity will be considered and random-effects model will be used to analyze, and if *I*^2^ < 50%, substantial heterogeneity will be considered to be absent and fixed-effects model will be used to analyze. Finally, subgroup analysis will be performed according to the different characteristics of RCTs to analyze the results with heterogeneity in order to find an explanation for the heterogeneity.

### 2.12. Sensitivity Analysis

If necessary, sensitivity analysis will be used to assess the effect of each study on random effects model. The exclusion method was used to analyze the sensitivity of the overall combined effect of all outcome measures. That is, each RCT will be excluded and the remaining RCTs will be reanalyzed to determine the stability of the results. Results will be considered stable if the combined effects shown by the results have not changed qualitatively.

### 2.13. Outcome Quality Analysis

The GRADE rating scale will be used in the quality assessment of the outcome measures [29–32]. The GRADE rating scale will be assessed by two independent investigators to assess the quality of the outcomes to make findings about the quality of the evidence. Quality assessments included risk of bias, inconsistency, indirectness, imprecision, publication bias, the effect value is very large, dose effect relationship, and negative bias. The quality of evidence will be rated as high, moderate, low, or very low.

### 2.14. Ethical approval and Dissemination

The final report of this systematic review will be published in a peer-reviewed scientific journal, and the dataset will be made freely available.

### 2.15. Amendments

If the protocol is modified, the change, the rationale, and the date of any amendment will be described in the final report.

## 3. Discussion

As the incidence of cardiovascular disease increases year by year, hyperlipidemia has become a public health problem affecting global health. Oral drugs for hyperlipidemia include statins, fibrates, and ezetimibe, but long-term drug therapy may cause various adverse reactions such as abnormal metabolism, liver function damage, and even rhabdomyolysis [21]. In recent years, many studies have shown that DSD plays an important role in controlling blood pressure, improving lipid metabolism, reducing the occurrence of atherosclerosis, and improving the quality of life of patients with cardiovascular diseases [15–19].

However, there is still a lack of systematic review on the efficacy and safety of DSD in the treatment of hyperlipidemia. Therefore, we aimed to summarize RCTs of DSD combined with CT or alone in the treatment of hyperlipidemia to provide sufficient evidence for the clinical efficacy of DSD in the treatment of hyperlipidemia. We will discuss further the limitations and prospects of DSD in the treatment of hyperlipidemia. The results of this meta-analysis may provide new perspectives for the clinical treatment of hyperlipidemia based on evidence-based medicine and will also help promote the development of TCM and the formulation of clinical guidelines.

## Figures and Tables

**Table 1:** Search strategy in PubMed.

Search	Query	Items found
#1	Clinical controlled experiments (MeSH terms)	
#2	Clinical observations (title/abstract)	
#3	Danshen decoction (title/abstract)	
#4	Danshen Yin (title/abstract)	
#5	Hypercholesterolemia (title/abstract)	
#6	Hypertriglyceridemia (title/abstract)	
#7	Blood lipids (title/abstract)	
#8	Triglycerides (title/abstract)	
#9	Cholesterol (title/abstract)	
#10	Hyperlipidemia (title/abstract)	
#11	High-density lipoprotein cholesterol (title/abstract)	
#12	Low-density lipoprotein cholesterol (title/abstract)	
#13	HDL-C (MeSH)	
#14	#1 OR #2 OR #3 OR #4 OR #5 OR #6 OR #7 OR #8 OR #9 OR #10 OR #11 OR #12 OR #13	

## Data Availability

All data are available from the corresponding author.

## Abbreviations

apoA1: Apolipoprotein A1
apoB: Apolipoprotein B
CER: Clinical effective rate
CI: Confidence interval
CVD: Cardiovascular disease
DSD: Danshen decoction
HDL-C: High-density lipoprotein cholesterol
HF: Heart failure
hs-CRP: Hypersensitive C-reactive protein
LDL-C: Low-density lipoprotein cholesterol
LP(a): Lipoprotein(a)
MD: Mean difference
PRISMA: The Preferred Reporting Items for Systematic Reviews and Meta-Analyses
RCT: Randomized-controlled trial
RR: Risk ratio
SMD: Standardized mean difference
TC: Serum total cholesterol
TG: Triglyceride
TCM: Traditional Chinese medicine.

## Supplementary Materials

Supplementary Materials The PRISMA-P checklist is used as supplementary material, all items are checked step by step, and the page numbers of all items are noted.
